# Differential Expression of Enzymes Associated with Serine/Glycine Metabolism in Different Breast Cancer Subtypes

**DOI:** 10.1371/journal.pone.0101004

**Published:** 2014-06-30

**Authors:** Sang Kyum Kim, Woo Hee Jung, Ja Seung Koo

**Affiliations:** Department of Pathology, Severance Hospital, Brain Korea 21 PLUS Project for Medical Science, Yonsei University College of Medicine, Seoul, South Korea; University of Central Florida, United States of America

## Abstract

**Purpose:**

Glycine and serine are well-known, classic metabolites of glycolysis. Here, we profiled the expression of enzymes associated with serine/glycine metabolism in different molecular subtypes of breast cancer and discuss their potential clinical implications.

**Methods:**

We used western blotting and immunohistochemistry to examine five serine-/glycine-metabolism–associated proteins (PHGDH, PSAT, PSPH, SHMT, and GLDC) in six breast cancer cell lines and 709 breast cancer cases using tissue microarray (TMA).

**Results:**

PHGDH and PSPH, associated with serine metabolism, were highly expressed in the TNBC cells. GLDC, associated with glycine metabolism, was highly expressed in HER-2-positive MDA-MB-453 and TNBC-related MDA-MB-435S. TMA showed that the TNBC-type breast cancer tissues highly expressed PHGDH, PSPH, and SHMT1, but not the luminal-A-type tissues (p<0.001). PSPH and SHMT1 expression in the tumor stroma of HER-2-type cancers was the highest, but the luminal-A tissues showed the lowest expression (p<0.001). GLDC was most frequently expressed in cancer cells and stroma of the HER-2-positive cancers and least frequently in TNBC (p<0.001). By Cox multivariate analysis, tumor PSPH positivity (hazard ratio [HR]: 2.068, 95% confidence interval [CI]: 1.049–4.079, p = 0.036), stromal PSPH positivity (HR: 2.152, 95% CI: 1.107–4.184, p = 0.024), and stromal SHMT1 negativity (HR: 2.142, 95% CI: 1.219–3.764, p = 0.008) were associated with short overall survival.

**Conclusions:**

Expression of serine-metabolism–associated proteins was increased in TNBC and decreased in the luminal-A cancers. Expression of glycine-metabolism–associated proteins was high in the tumor and stroma of HER-2-positive cancers.

## Introduction

The “Warburg effect” explains a much higher rate of glycolysis followed by fermentation in the cancer cell mitochondria. According to the Warburg effect, glycolytic intermediates, especially those involved in glycine and serine metabolism, rapidly accumulate in cancer cells [1]. In serine biosynthesis, 3-phosphoglycerate (3PG) produced by glycolysis is oxidized to 3-phosphohydroxypyruvate (pPYR) by phosphoglycerate dehydrogenase (PHGDH), and then pPYR is transaminated to phosphoserine (pSER) by phosphoserine aminotransferase (PSAT). Finally, pSER is dephosphorylated to serine by phosphoserine phosphatase (PSPH). In glycine metabolism, glycine is converted to methylenetetrahydrofolate by glycine decarboxylase (GLDC). On the other hand, serine hydroxymethyltransferase (SHMT) converts serine to glycine reversibly, linking the respective metabolic pathways. According to previous reports, these enzymes are highly expressed in several human tumors: PHGDH in breast cancer and melanoma [2], [3] and GLDC in lung cancer [4]. Therefore, they likely play important roles in tumorigenesis [2], [3], [4], [5].

Breast cancer is heterogeneous because it shows disparate clinical, histopathological, and genetic characteristics. Classification of breast cancer is actively underway to identify common characteristics amongst the various subtypes. Breast cancer subtypes include the luminal-A, luminal-B, HER-2, normal-breast-like, and basal-like types defined by gene-profiling analyses [6], [7]. Another type, which is negative for estrogen receptor (ER), progesterone receptor (PR), and HER-2—all used as biomarkers in breast cancer treatment—is defined as the triple-negative breast cancer (TNBC) [8].

We believe that metabolic characteristics specific to each subtype could present distinctive hallmarks because each type differs histopathologically, clinically, therapeutically, and prognostically. High levels of glucose transporter 1 (GLUT-1) and carbonic anhydrase 9 (CA9), glycolysis-associated enzymes, were found in the basal-like type and TNBC subtype [9], [10], but expression of glutaminolysis-associated proteins was increased in the HER-2 type [11]. However, few studies have established expression profiling of enzymes associated with serine/glycine metabolism in breast cancer to support a molecular relationship between subtypes and their respective metabolic characteristics. In this study, we profiled the expression of several enzymes associated with serine/glycine metabolism and explored their potential clinical significance. These included PHGDH, PSAT, PSPH, SHMT, and GLDC.

## Materials and Methods

### Cells and cell culture

Six breast cancer cell lines, MCF-7, MDA-MB-361, MDA-MB-453, MDA-MB-435S, MDA-MB-231, and MDA-MB-468 (all from the American Type Culture Collection), were examined. MCF-7 was maintained in Dulbecco's Modified Eagle's Medium/Nutrient Mixture F12 (DMEM/F12; Gibco) without phenol red, but supplemented with 10 µg/mL insulin (Sigma), 10% fetal bovine serum (Gibco), and 1% penicillin/streptomycin (Gibco). The other cells were maintained in DMEM/F12 containing 10% fetal bovine serum and 1% penicillin/streptomycin. All cells were cultured at 37°C in a humidified atmosphere containing 5% CO_2_.

### Western blotting

For western blotting, ∼8×10^5^ cells were seeded in 60-mm dishes. After 24 h, cells were washed twice with cold phosphate-buffered saline and lysed in the lysis buffer (50 mM Tris-HCL [pH 7.9], 100 mM NaCl, 1 mM EDTA, 2% SDS, 0.1 mM EDTA, and 0.1 mM EGTA) containing a protease and phosphatase inhibitor cocktail (Thermo Scientific). Twenty micrograms of protein was treated with Laemmli sample buffer, heated at 100°C for 5 min, and resolved on 8% or 12% sodium dodecyl sulfate-polyacrylamide gel electrophoresis. Gels were electroblotted onto nitrocellulose membranes (GE Healthcare life-Sciences), which were blocked in 5% non-fat dry milk in TBS-T, and incubated with antibodies for PHGDH, PSAT1, PSPH, SHMT1, GLDC (all at 1∶1000, obtained from Abcam), or β-actin (1∶2000, Sigma) overnight at 4°C. Membranes were subsequently washed thrice in TBS-T and probed with peroxidase-conjugated goat anti-mouse IgG (1∶2000, Santa Cruz) for 1 h at room temperature. Membranes were washed again and developed using a chemiluminescent reagent (ECL; GE Healthcare Life Sciences, Inc.). Band densities were measured using TINA imaging software (Raytest, Straubenhardt, Germany).

### Patient selection

The institutional review board of Yonsei University Severance Hospital approved this retrospective study. The study population included 709 patients who had been diagnosed histopathologically with invasive ductal carcinoma before tumor excision at Yonsei University Severance Hospital from 2002 to 2006. Patients who had received hormone therapy or chemotherapy before surgery were excluded. This study was approved by the Institutional Review Board (IRB) of Yonsei University Severance Hospital. IRB waived the informed consent form from patients. Patient records/information was anonymized and de-identified prior to analysis. Patients' tissue samples were fixed in 10% buffered formalin and embedded in paraffin. Archival tissues stained with hematoxylin and eosin (H&E) were reviewed by three breast pathologists (SK Kim, WH Jung, and JS Koo). Histopathology grading was done according to the Nottingham grading system [12]. Patients' clinicopathological characteristics included age at initial diagnosis, lymph-node metastasis, tumor recurrence, distant metastasis, and survival.

### Tissue microarray

A random area was selected on an H&E-stained tissue slide and the corresponding area was marked on the surface of the corresponding paraffin-embedded tissue block. The selected area was punctured using a biopsy needle, and a 3-mm tissue core was extracted and transferred onto a 6×5 recipient block. Two tissue cores were extracted to minimize extraction bias. Each tissue core was assigned a unique tissue microarray location number that was linked to a database recording other clinicopathological data.

### Immunohistochemistry

Antibodies used are shown in Table 1. Immunostaining was performed using formalin-fixed, paraffin-embedded (FFPE) tissue sections. Briefly, 5-µm-thick sections were cut using a microtome, transferred onto adhesive slides, and dried at 62°C for 30 min. After incubation with primary antibodies, immunodetection was performed using biotinylated anti-mouse immunoglobulin, followed by streptavidin-conjugated peroxidase from a streptavidin–biotin kit. 3,3′-Diaminobenzidine was used as the chromogen substrate. Incubation with the primary antibody was omitted for the negative controls. Positive control tissue samples were used as recommended by the manufacturer. Slides were counterstained with Harris hematoxylin.

**Table 1:** Source, clone, and dilution of antibodies.

Antibody	Company	Clone	Dilution
*serine/glycine metabolism related proteins*			
PHGDH	Abcam, Cambridge, UK	Polyclonal	1∶100
PSAT1	Abcam, Cambridge, UK	Polyclonal	1∶100
PSPH	Abcam, Cambridge, UK	Polyclonal	1∶100
SHMT	Abcam, Cambridge, UK	Polyclonal	1∶100
GLDC	Abcam, Cambridge, UK	Polyclonal	1∶100
*molecular subtype related proteins*			
ER	Thermo Scientific, San Siego, CA, USA	SP1	1∶100
PR	DAKO, Glostrup, Denmark	PgR	1∶50
HER-2	DAKO, Glostrup, Denmark	Polyclonal	1∶1500
Ki-67	Abcam, Cambridge, UK	MIB	1∶1000

### Interpretation of immunohistochemical staining

Light microscopy was used to visualize the expression of immunohistochemical markers. Pathological status, including ER, PR, and HER-2 positivity were gathered using patients' pathologic reports. A cut-off value of >1% positively stained nuclei was used to define ER and PR positivity [13]. HER-2 staining was analyzed according to guidelines by the American Society of Clinical Oncology (ASCO)/College of American Pathologists (CAP): 0 =  no immunostaining; 1+ =  weak incomplete membranous staining of <10% of tumor cells; 2+ =  complete membranous staining, either uniform or weak, of ≥10% of tumor cells; and 3+ =  uniform intense membranous staining of ≥30% of tumor cells [14]. HER-2 immunostaining was considered positive when strong (3+) membranous staining was observed whereas cases with 0 to 1+ were regarded as negative. Cases showing 2+ HER-2 expression were further examined by fluorescent *in situ* hybridization (FISH) for HER-2 amplification.

For measuring immunostaining intensity, we divided breast cancers into four groups as follows: 0 (negative), 1 (weakly positive), 2 (moderately positive), and 3 (strongly positive). For measuring the proportion of stained cells, we divided breast cancers into three groups as follows: 0 (negative), 1 (positive <30%), and 2 (positive >30%). Immunohistochemical values for GLDC, PSAT, PSPH, PHGDH, and SHMT were calculated by multiplying immunostaining intensity to the proportion of stained cells. The final score after multiplication was classified as follows: 0–1 as negative and 2–6 as positive [15]. Ki-67 labeling index (LI) was defined as the percentage of cells with positive nuclei to total number of cancerous cells.

### Tumor phenotype classification

Breast cancer phenotypes were classified according to immunohistochemical results for ER, PR, HER-2, and Ki-67 and HER-2 FISH results as follows [16]: *luminal A type*: ER and/or PR positive, HER-2 negative, and Ki-67 LI <14%; *luminal B type*: (HER-2 negative) ER and/or PR positive, HER-2 negative, and Ki-67 LI ≥14%, (HER-2 positive) ER and/or PR positive, HER-2 overexpressed and/or amplified; *HER-2 overexpression type*: ER and PR negative and HER-2 overexpressed and/or amplified; and *TNBC type*: ER, PR, and HER-2 negative.

### FFPE tissue microdissection and protein extraction

Hematoxylin-stained, uncovered slides were prepared using FFPE tissue blocks of five breast cancer cases of each molecular subtype. Tumor components or the corresponding stroma was then captured using laser microdissection (LMD 6500, Leica, Wetzlar, Germany). Microdissected FFPE tissues were deparaffinized in xylene and rehydrated in a graded series of alcohol. Total protein was extracted from the captured microdissected FFPE material using the Qproteome FFPE Tissue Kit (Qiagen, Hilden, Germany). Samples were mixed with the FFPE extraction buffer EXB Plus (100 µl per sample), incubated at 100°C for 20 min, at 80°C for 2 h, and finally centrifuged at 14,000 *g* at 4°C for 15 min. The resultant supernatants were measured by the Bradford assay (Bio-Rad, USA) to determine protein concentration.

### Statistical analyses

Data were analyzed using the SPSS software (Version 12.0; SPSS Inc., Chicago, IL, USA) for Microsoft Windows. Statistical significance was established by the Student's *t* and Fisher's exact tests for continuous and categorical variables, respectively. A corrected *p*-value and the Bonferroni method were used for multiple comparisons. Statistical significance was when p<0.05. Kaplan–Meier survival curves and log-rank statistics were used to evaluate time to tumor recurrence and overall survival. Multivariate regression analysis was performed using the Cox proportional-hazards model.

## Results

### Detection of enzymes associated with serine/glycine metabolism in cell lines

**Figure 1:**
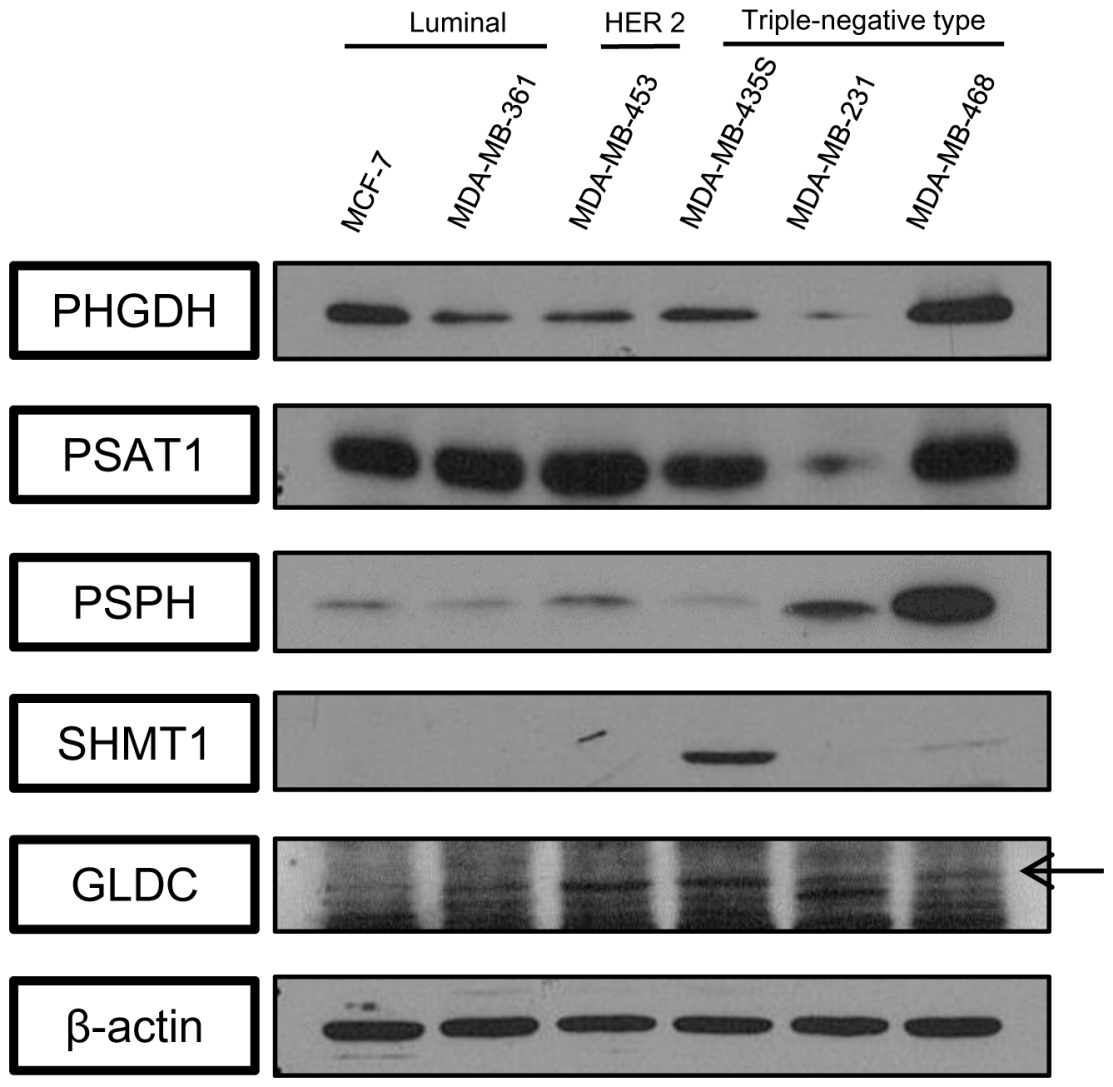
Expression of enzymes associated with serine/glycine metabolism in breast cancer cells detected by western blotting. PHGDH and PSPH levels were increased in the TNBC cell lines and the GLDC levels was increased in MDA-MB-453 of the HER-2 subtype and MDA-MB-435S of the TNBC subtype. SHMT1 was highly expressed in the HER-2 subtype and MDA-MB-435S cells.

Western blotting of enzymes associated with serine/glycine metabolism in six human breast cancer cell lines is presented in Figure 1. The density of each protein was calculated relative to β-actin and assessed in relation to the molecular subtypes of the tested cell lines: MCF-7 and MDA-MB-361 representing the luminal type; MDA-MB-453, HER-2 type; and MDA-MB-453S, MDA-MB-231, and MDA-MB-468, TNBC type. We confirmed that these proteins were expressed in cell lines of the luminal, HER-2, and TNBC types.

### Detection of enzymes associated with serine/glycine metabolism in patients' specimens

**Figure 2:**
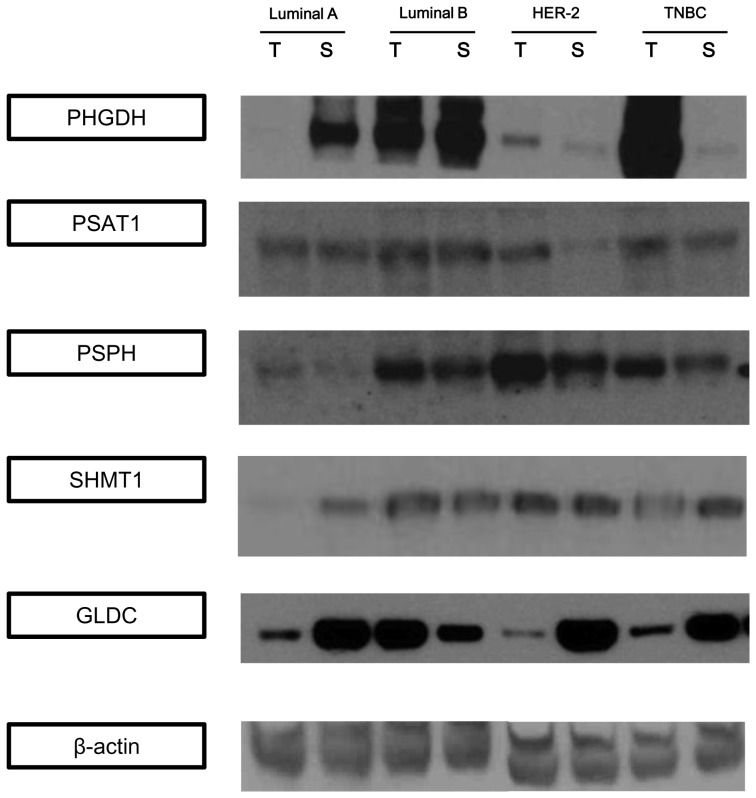
Western blotting detection of enzymes associated with serine/glycine metabolism in breast cancer tissues. PHGDH levels were the highest in the epithelial component of the TNBC and the lowest in the epithelial component of the luminal-A subtype. PSPH and SHMT1 expressions were decreased in the luminal-A subtype. GLDC expression was the highest in the tumor epithelial compartment of the luminal-B subtype and in the stromal compartment of the HER-2 subtype.

Next, expression of the abovementioned proteins was examined in patients' specimens and correlated to the corresponding molecular subtypes. Each tumor tissue was divided into epithelial and the corresponding stromal components by laser microdissection. Protein expression profiles were compared between the epithelial and stromal components in each cancer subtype (Figure 2). All serine-metabolism–associated enzymes were expressed to a greater extent in the epithelial than in the stromal component of the HER-2 and TNBC types. GLDC was expressed to a greater extent in the epithelial rather than in the stromal component of the luminal-B, HER-2, and TNBC types; however, the opposite pattern was observed in the luminal-A cancers.

### Patients' clinicopathological characteristics

Breast cancer tissues included in the TMA analysis were classified into luminal A (297 cases, 41.9%), luminal B (168 cases, 23.7%), HER-2 (70 cases, 9.9%), or TNBC (174 cases, 24.5%; Table 2). Notably, the TNBC type demonstrated higher histopathological grading (p<0.001), higher T staging (p = 0.014), and higher Ki-67 LI (p<0.001) than the other subtypes. Whereas, the HER-2 type occurred in older patients (p = 0.011), more frequently recurred, and resulted in higher death rates (p = 0.001) than the other types (Table 2).

**Table 2:** Patients' clinicopathological characteristics according to breast cancer phenotype.

Parameters	Total (n = 709) (%)	Luminal A (n = 297) (%)	Luminal B (n = 168) (%)	HER-2 (n = 70) (%)	TNBC (n = 174) (%)	*P*-value
Age (yr, mean ±SD)	49.7±10.9	50.5±10.5	48.4±10.0	52.4±10.0	48.2±12.5	**0.011**
Histologic grade						<0.001
I	119 (16.7)	90 (30.3)	19 (11.3)	1 (1.4)	7 (4.0)	
II	360 (50.6)	179 (60.3)	92 (54.8)	36 (51.4)	53 (30.5)	
III	232 (32.6)	28 (9.4)	57 (33.9)	33 (47.1)	114 (65.5)	
Tumor stage						**0.014**
T1	346 (48.7)	162 (54.5)	86 (51.2)	30 (42.9)	66 (37.9)	
T2	350 (49.2)	127 (42.8)	80 (47.6)	39 (55.7)	104 (59.8)	
T3	15 (2.1)	8 (2.7)	2 (1.2)	1 (1.4)	4 (2.3)	
Nodal stage						**0.055**
N0	420 (59.1)	170 (57.2)	92 (54.8)	42 (60.0)	115 (66.1)	
N1	188 (26.4)	86 (29.0)	43 (25.6)	13 (18.6)	45 (25.9)	
N2	64 (9.0)	26 (8.8)	18 (10.7)	10 (14.3)	10 (5.7)	
N3	39 (5.5)	15 (5.1)	15 (8.9)	5 (7.1)	4 (2.3)	
Estrogen receptor status						**<0.001**
Negative	254 (35.7)	5 (1.7)	5 (3.0)	70 (100.0)	174 (100.0)	
Positive	457 (64.3)	292 (98.3)	163 (97.0)	0 (0.0)	0 (0.0)	
Progesterone receptor status						**<0.001**
Negative	339 (47.7)	48 (16.2)	48 (28.6)	69 (98.6)	174 (100.0)	
Positive	372 (52.3)	249 (83.8)	120 (71.4)	1 (1.4)	0 (0.0)	
HER-2 status						**<0.001**
0	262 (36.8)	107 (36.0)	24 (14.3)	0 (0.0)	129 (74.1)	
1+	184 (25.9)	119 (40.1)	33 (19.6)	0 (0.0)	32 (18.4)	
2+	141 (19.8)	71 (23.9)	41 (24.4)	16 (22.9)	13 (7.5)	
3+	124 (17.4)	0 (0.0)	70 (41.7)	54 (77.1)	0 (0.0)	
Ki-67 LI (%, mean ±SD)	17.4±18.4	4.7±3.7	19.5±12.4	19.7±12.9	36.1±22.9	**<0.001**
Tumor recurrence	63 (8.9)	14 (4.7)	13 (7.7)	12 (17.1)	24 (13.8)	**0.001**
Patients' death	59 (8.3)	12 (4.0)	12 (7.1)	12 (17.1)	23 (13.2)	**<0.001**
Duration of clinical follow-up (months, mean ±SD)	69.9±31.2	72.7±29.6	70.1±30.1	64.9±34.1	67.0±33.5	0.127

TNBC, triple negative breast cancer.

### Expression profiling of metabolism-associated enzymes in relation to cancer phenotypes

**Figure 3:**
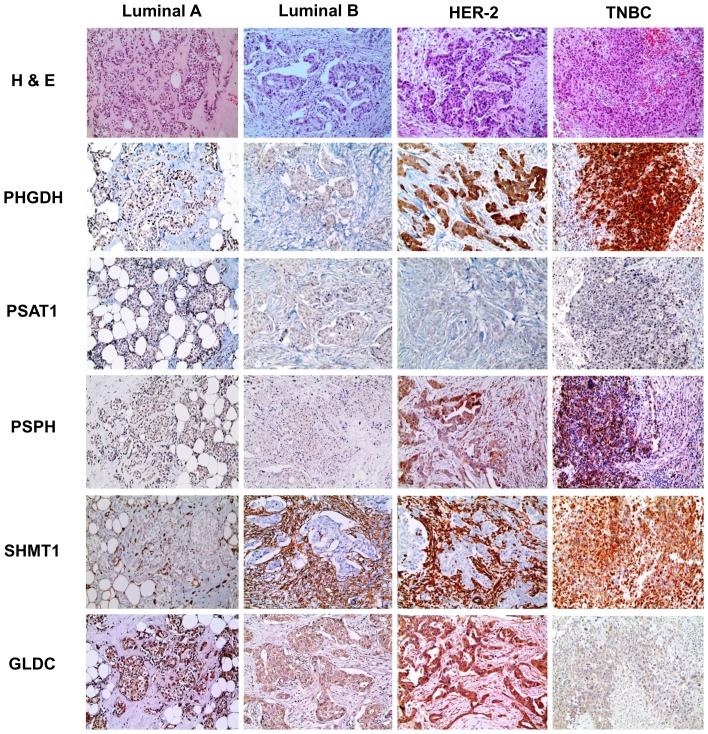
Expression of enzymes associated with serine/glycine metabolism in different molecular breast cancer subtypes. PHGDH levels were increased in the epithelial component of the HER-2 and TNBC subtypes. PSPH was increased in the tumor epithelium of the TNBC subtype and in the HER-2 stroma. SHMT1 expression was high in the epithelial component of the TNBC subtype and in the stromal component of the luminal-B and HER-2 subtypes. GLDC was high in the epithelial and stromal components of the HER-2 subtype.

**Figure 4:**
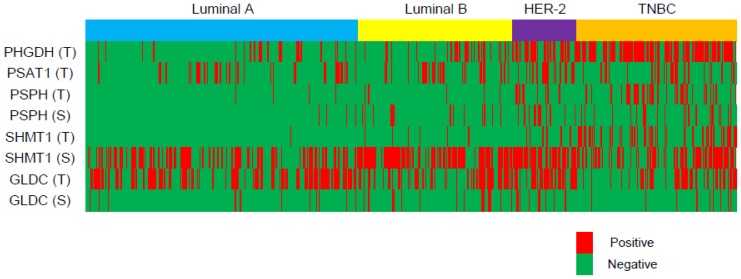
Expression heat map showing levels of enzymes associated with serine/glycine metabolism according to breast cancer molecular subtype.

Serine-/glycine-metabolism–associated proteins were investigated in cancer tissues by immunohistochemistry (Figure 3, Figure 4 and Table 3). PHGDH, PSPH, and SHMT1 were highly expressed in the epithelial component of the TNBC subtype. However, expression was the lowest in the luminal-A subtype (p<0.001). Similarly, the stromal component expressed the highest PSPH and SHMT1 levels in the HER-2, but the lowest in the luminal-A subtype (p<0.001).

**Table 3:** Expression of metabolism-associated enzymes according to breast cancer phenotype.

Parameters	Total (n = 709) (%)	Luminal A (n = 297) (%)	Luminal B (n = 168) (%)	HER-2 (n = 70) (%)	TNBC (n = 174) (%)	*P*-value
PHGDH in tumor						**<0.001**
Negative	452 (63.8)	253 (85.2)	124 (73.8)	33 (47.1)	42 (24.1)	
Positive	132 (75.9)	44 (14.8)	44 (26.2)	37 (52.9)	132 (75.9)	
PSAT1 in tumor						0.061
Negative	526 (74.2)	229 (77.1)	126 (75.0)	43 (61.4)	128 (73.6)	
Positive	183 (25.8)	68 (22.9)	42 (25.0)	27 (38.6)	46 (26.4)	
PSPH in tumor						**<0.001**
Negative	611 (86.2)	284 (95.6)	157 (93.5)	55 (78.6)	115 (66.1)	
Positive	98 (13.8)	13 (4.4)	11 (6.5)	15 (21.4)	59 (33.9)	
PSPH in stroma						**<0.001**
Negative	625 (88.2)	287 (96.6)	141 (83.9)	54 (77.1)	143 (82.2)	
Positive	84 (11.8)	10 (3.4)	27 (16.1)	16 (22.9)	31 (17.8)	
SHMT1 in tumor						**<0.001**
Negative	598 (84.3)	284 (95.6)	154 (91.7)	59 (84.3)	101 (58.0)	
Positive	111 (15.7)	13 (4.4)	14 (8.3)	11 (15.7)	73 (42.0)	
SHMT1 in stroma						**<0.001**
Negative	310 (43.7)	156 (52.5)	51 (30.4)	19 (27.1)	84 (48.3)	
Positive	399 (56.3)	141 (47.5)	117 (69.6)	51 (72.9)	90 (51.7)	
GLDC in tumor						**<0.001**
Negative	370 (52.2)	138 (46.5)	97 (57.7)	26 (37.1)	109 (62.6)	
Positive	339 (47.8)	159 (53.5)	71 (42.3)	44 (62.9)	65 (37.4)	
GLDC in stroma						**<0.001**
Negative	621 (87.6)	271 (91.2)	135 (80.4)	53 (75.7)	162 (93.1)	
Positive	88 (12.4)	26 (8.8)	33 (19.6)	17 (24.3)	12 (6.9)	

TNBC, triple negative breast cancer.

GLDC ranked the highest, in both the epithelial and stromal components, in the HER-2 subtype, but the lowest in TNBC (p<0.001).

### Correlations between protein expression and patients' clinicopathological characteristics

After assessing expression profiles of the tested proteins, we examined whether expression profiles were correlated with patients' clinicopathological characteristics (Table 4 and Table 5). High levels of PHGDH, PSPH, SHMT1 in tumor and PSPH in tumor stroma were correlated with high histological grading (p<0.001), ER negativity (p<0.001), PR negativity (p<0.001), and high Ki-67 LI (p<0.001). High levels of SHMT1 and GLDC in the stromal component were correlated with HER-2 positivity (p<0.001). Expression of stromal PSPH was associated with higher T staging (p = 0.048), whereas expression of tumor GLDC was associated with lower T staging (p = 0.008) and lower Ki-67 LI (p = 0.008).

**Table 4:** Correlations between expression of serine-metabolism–associated enzymes and clinicopathological characteristics.

Parameters	PHGDH in tumor			PSAT1 in tumor			PSPH in tumor			PSPH in stroma		
	Negative n = 452 (%)	Positive n = 257 (%)	*P*-value*	Negative n = 526 (%)	Positive n = 183 (%)	*P*-value*	Negative n = 611 (%)	Positive n = 98 (%)	*P*-value*	Negative n = 625 (%)	Positive n = 84 (%)	*P*-value*
Age (yr, mean ±SD)	49.6±10.5	49.7±11.6	7.656	49.6±10.7	49.8±11.6	6.504	49.7±10.8	49.7±11.7	7.896	49.8±10.9	48.9±10.7	4.112
Histologic grade			**<0.001**			1.608			**<0.001**			**0.001**
I/II	353 (78.1)	124 (48.2)		361 (68.6)	116 (63.4)		437 (71.5)	40 (40.8)		434 (69.4)	43 (51.2)	
III	99 (21.9)	133 (51.8)		165 (31.4)	67 (36.6)		174 (28.5)	58 (59.2)		191 (30.6)	41 (48.8)	
ER			**<0.001**			0.392			**<0.001**			**<0.001**
Negative	81 (17.9)	173 (67.3)		177 (33.7)	77 (42.1)		179 (29.3)	75 (76.5)		204 (32.6)	50 (59.5)	
Positive	371 (82.1)	84 (32.7)		349 (66.3)	106 (57.9)		432 (70.7)	23 (23.5)		421 (67.4)	34 (40.5)	
PR			**<0.001**			0.688			**<0.001**			**<0.001**
Negative	150 (33.2)	189 (73.5)		241 (45.8)	98 (53.6)		258 (42.2)	81 (82.7)		283 (45.3)	56 (66.7)	
Positive	302 (66.8)	68 (26.5)		285 (54.2)	85 (46.4)		353 (57.8)	17 (17.3)		342 (54.7)	28 (33.3)	
HER-2			6.784			0.360			5.568			0.120
Negative	359 (79.4)	202 (78.6)		426 (81.0)	135 (73.8)		482 (78.9)	79 (80.6)		503 (80.5)	58 (69.0)	
Positive	93 (20.6)	55 (21.4)		100 (19.0)	48 (26.2)		129 (21.1)	19 (19.4)		122 (19.5)	26 (31.0)	
Tumor stage			0.408			0.160			1.232			**0.048**
T1	232 (51.3)	112 (43.6)		269 (51.1)	75 (41.0)		303 (49.6)	41 (41.8)		315 (50.4)	29 (34.5)	
T2/T3	220 (48.7)	145 (56.4)		257 (48.9)	108 (59.0)		308 (50.4)	57 (58.2)		310 (49.6)	55 (65.5)	
Nodal stage			0.904			3.072			0.936			5.584
N0	257 (56.9)	162 (63.0)		316 (60.1)	103 (56.3)		354 (57.9)	65 (66.3)		371 (59.4)	48 (57.1)	
N1/N2/N3	195 (43.1)	95 (37.0)		210 (39.9)	80 (43.7)		257 (42.1)	33 (33.7)		254 (40.6)	36 (42.9)	
Ki-67 LI (%, mean ±SD)	11.0±11.7	28.6±22.4	**<0.001**	18.0±19.3	15.5±15.6	0.912	15.7±17.5	27.8±20.5	**<0.001**	16.1±17.9	27.3±19.3	**<0.001**
Tumor recurrence			2.184			2.328			0.808			1.192
Absent	416 (92.0)	230 (89.5)		483 (91.8)	163 (89.1)		561 (91.8)	85 (86.7)		573 (91.7)	73 (86.9)	
Present	36 (8.0)	27 (10.5)		43 (8.2)	20 (10.9)		50 (8.2)	13 (13.3)		52 (8.3)	11 (13.1)	
Death			0.536			0.696			0.168			0.088
Survival	421 (93.1)	229 (89.1)		488 (92.8)	162 (88.5)		566 (92.6)	84 (85.7)		579 (92.6)	71 (84.5)	
Death	31 (6.9)	28 (10.9)		38 (7.2)	21 (11.5)		45 (7.4)	14 (14.3)		46 (7.4)	13 (15.5)	

*p-value was corrected by the Bonferroni method.

**Table 5:** Correlations between expression of enzymes associated with glycine metabolism and clinicopathological characteristics.

Parameters	SHMT1 in tumor			SHMT1 in stroma			GLDC in tumor			GLDC in stroma		
	Negative n = 598 (%)	Positive n = 111 (%)	*P*-value*	Negative n = 310 (%)	Positive n = 399 (%)	*P*-value*	Negative n = 370 (%)	Positive n = 339 (%)	*P*-value*	Negative n = 621 (%)	Positive n = 88 (%)	*P*-value*
Age (yr, mean ±SD)	50.2±10.9	46.9±10.7	**0.032**	49.8±11.7	49.6±10.3	6.448	48.5±11.0	50.9±10.7	**0.024**	49.6±10.9	50.0±10.8	6.072
Histologic grade			**<0.001**			1.168			4.600			0.416
I/II	431 (72.1)	46 (41.4)		218 (70.3)	259 (64.9)		245 (66.2)	232 (68.4)		426 (68.6)	51 (58.0)	
III	167 (27.9)	65 (58.6)		92 (29.7)	140 (35.1)		125 (33.8)	107 (31.6)		195 (31.4)	37 (42.0)	
ER			**<0.001**			6.024			2.176			6.496
Negative	164 (27.4)	90 (81.1)		109 (35.2)	145 (36.3)		140 (37.8)	114 (33.6)		224 (36.1)	30 (34.1)	
Positive	434 (72.6)	21 (18.9)		201 (64.8)	254 (63.7)		230 (62.2)	225 (66.4)		397 (63.6)	58 (65.9)	
PR			**<0.001**			5.200			**0.024**			6.568
Negative	253 (42.3)	68 (77.5)		145 (46.8)	194 (48.6)		197 (53.2)	142 (41.9)		298 (48.0)	41 (46.6)	
Positive	345 (57.7)	25 (22.5)		165 (53.2)	205 (51.4)		173 (46.8)	197 (58.1)		323 (52.0)	47 (53.4)	
HER-2			1.024			**<0.001**			0.128			**<0.001**
Negative	467 (78.1)	94 (84.7)		269 (86.8)	292 (73.2)		306 (82.7)	255 (75.2)		506 (81.5)	55 (62.5)	
Positive	131 (21.9)	17 (15.3)		41 (13.2)	107 (26.8)		64 (17.3)	84 (24.8)		115 (18.5)	33 (37.5)	
Tumor stage			0.392			2.312			**0.008**			4.560
T1	300 (50.2)	44 (39.6)		143 (46.1)	201 (50.4)		158 (42.7)	186 (54.9)		304 (49.0)	40 (45.5)	
T2/T3	298 (49.8)	67 (60.4)		167 (53.9)	198 (49.6)		212 (57.3)	153 (45.1)		317 (51.0)	48 (54.5)	
Nodal stage			0.736			6.064			5.176			0.216
N0	345 (57.7)	74 (66.7)		181 (58.4)	238 (59.6)		222 (60.0)	197 (58.1)		377 (60.7)	42 (47.7)	
N1/N2/N3	253 (42.3)	37 (33.3)		129 (41.6)	161 (40.4)		148 (40.0)	142 (41.9)		244 (39.3)	46 (52.3)	
Ki-67 LI (%, mean ± SD)	14.5±16.0	32.9±22.6	**<0.001**	16.4±19.5	18.1±17.6	1.800	19.6±19.7	14.9±16.7	**0.008**	17.3±18.8	18.0±15.8	5.808
Tumor recurrence			2.200			0.880			0.136			5.504
Absent	548 (91.6)	98 (88.3)		276 (89.0)	370 (92.7)		328 (88.6)	318 (93.8)		567 (91.3)	79 (89.8)	
Present	50 (8.4)	13 (11.7)		34 (11.0)	29 (7.3)		42 (11.4)	21 (6.2)		54 (8.7)	9 (10.2)	
Death			1.504			0.104			1.392			8.000
Survival	552 (92.3)	98 (88.3)		275 (88.7)	375 (94.0)		334 (90.3)	316 (93.2)		569 (91.6)	81 (92.0)	
Death	46 (7.7)	13 (11.7)		35 (11.3)	24 (6.0)		36 (9.7)	23 (6.8)		52 (8.4)	7 (8.0)	

*p-value was corrected by the Bonferroni method.

### Correlation between expression profiles of proteins associated with serine or glycine metabolism and glycolysis-associated proteins

Previously, we reported differential expression patterns of glycolysis-associated enzymes, including Glut-1, CA9, and monocarboxylate transporter 4 (MCT4) in tissues derived from different breast cancer subtypes [10], [17]. Here, we compared expression profiles of these proteins with those associated with serine or glycine metabolism to identify a possible link between these metabolic pathways in the context of the breast cancer (Table 6 and Table 7). By correlation analyses, we found that expression of glycolysis-associated and serine-/-glycine-metabolism–associated enzymes were correlated in separate tumor components, tumoral and stromal, as follows: tumoral Glut-1 and CA9 and tumoral PHGDH, PSPH, and SHMT1 (p<0.05); tumoral CA9, MCT4, and tumoral PSPH (p<0.001); stromal Glut-1 and tumoral PSPH (p = 0.018), stromal PSPH (p<0.001) and stromal GLDC (p<0.001); stromal CA9 and stromal PSPH (p<0.001), SHMT1 (p<0.001), and GLDC (p<0.001); stromal MCT4 and tumoral PHGDH (p<0.001), tumoral PSPH (p<0.001), stromal PSPH (p<0.001), stromal SHMT1 (p<0.001), and stromal GLDC (p<0.001).

**Table 6:** Correlations between expression of enzymes associated with serine/glycine metabolism and glycolysis.

Parameters	Glut-1 in tumor			CAIX in tumor			MCT4 in tumor		
	Negative n = 499 (%)	Positive n = 210 (%)	*P*-value*	Negative n = 499 (%)	Positive n = 210 (%)	*P*-value*	Negative n = 529 (%)	Positive n = 180 (%)	*P*-value*
PHGDH in tumor			**<0.001**			**<0.001**			0.132
Negative	361 (72.3)	91 (43.3)		349 (69.3)	106 (50.5)		350 (66.2)	102 (56.7)	
Positive	138 (27.7)	119 (56.7)		153 (30.7)	104 (49.5)		179 (33.8)	78 (43.3)	
PSAT1 in tumor			2.850			1.968			0.846
Negative	374 (74.9)	152 (72.4)		365 (73.1)	161 (76.7)		385 (72.8)	141 (78.3)	
Positive	125 (25.1)	58 (27.6)		134 (26.9)	49 (23.3)		144 (27.2)	39 (21.7)	
PSPH in tumor			**<0.001**			**<0.001**			**<0.001**
Negative	462 (92.6)	149 (71.0)		447 (89.6)	164 (78.1)		471 (89.0)	140 (77.8)	
Positive	37 (7.4)	61 (29.0)		52 (10.4)	46 (21.9)		58 (11.0)	40 (22.2)	
PSPH in stroma			0.120			2.784			0.240
Negative	449 (90.0)	176 (83.8)		437 (87.6)	188 (89.5)		474 (89.6)	151 (83.9)	
Positive	50 (10.0)	34 (16.2)		62 (12.4)	22 (10.5)		55 (10.4)	29 (16.1)	
SHMT1 in tumor			**<0.001**			**0.018**			0.216
Negative	456 (91.4)	142 (67.6)		434 (87.0)	164 (78.1)		455 (86.0)	143 (79.4)	
Positive	43 (8.6)	68 (32.4)		65 (13.0)	46 (21.9)		74 (14.0)	37 (20.6)	
SHMT1 in stroma			0.546			0.768			4.926
Negative	208 (41.7)	102 (48.6)		209 (41.9)	101 (48.1)		230 (43.5)	80 (44.4)	
Positive	291 (58.3)	108 (51.4)		290 (58.1)	109 (51.9)		299 (56.5)	100 (55.6)	
GLDC in tumor			1.332			**<0.001**			3.576
Negative	253 (50.7)	117 (55.7)		286 (57.3)	84 (40.0)		273 (51.6)	97 (53.9)	
Positive	246 (49.3)	93 (44.3)		213 (42.3)	126 (60.0)		256 (48.4)	83 (46.1)	
GLDC in stroma			0.144			4.746			2.922
Negative	428 (85.8)	193 (91.9)		436 (87.4)	185 (88.1)		466 (88.1)	155 (86.1)	
Positive	71 (14.2)	17 (8.1)		63 (12.6)	25 (11.9)		63 (11.9)	25 (13.9)	

*p-value was corrected by the Bonferroni method.

**Table 7:** Correlations between expression enzymes associated with serine/glycine metabolism and stromal expression of glycolysis-associated enzymes.

Parameters	Glut-1 in stroma			CAIX in stroma			MCT4 in stroma		
	Negative n = 693 (%)	Positive n = 16 (%)	*P*-value*	Negative n = 595 (%)	Positive n = 114 (%)	*P*-value*	Negative n = 409 (%)	Positive n = 300 (%)	*P*-value*
PHGDH in tumor			1.482			0.618			<0.001
Negative	444 (64.1)	8 (50.0)		387 (65.0)	65 (57.0)		297 (72.6)	155 (51.7)	
Positive	249 (35.9)	8 (50.0)		208 (35.0)	49 (43.0)		112 (27.4)	145 (48.3)	
PSAT1 in tumor			6.000			0.462			4.710
Negative	514 (74.2)	12 (75.0)		449 (75.5)	77 (67.5)		305 (74.6)	221 (73.7)	
Positive	179 (25.8)	4 (25.0)		146 (24.5)	37 (32.5)		104 (25.4)	79 (26.3)	
PSPH in tumor			**0.018**			1.410			**<0.001**
Negative	602 (86.9)	9 (56.3)		517 (86.9)	94 (82.5)		372 (91.0)	239 (79.7)	
Positive	91 (13.1)	7 (43.8)		78 (13.1)	20 (17.5)		37 (9.0)	61 (20.3)	
PSPH in stroma			**<0.001**			**<0.001**			**<0.001**
Negative	619 (89.3)	6 (37.5)		542 (91.1)	83 (72.8)		392 (95.8)	233 (77.7)	
Positive	74 (10.7)	10 (62.5)		53 (8.9)	31 (27.2)		17 (4.2)	67 (22.3)	
SHMT1 in tumor			1.788			4.476			0.558
Negative	586 (84.6)	12 (75.0)		503 (84.5)	95 (83.3)		353 (86.3)	245 (81.7)	
Positive	107 (15.4)	4 (25.0)		92 (15.5)	19 (16.7)		56 (13.7)	55 (18.3)	
SHMT1 in stroma			0.060			**<0.001**			**<0.001**
Negative	308 (44.4)	2 (12.5)		292 (49.1)	18 (15.8)		236 (57.7)	74 (24.7)	
Positive	385 (55.6)	14 (87.5)		303 (50.9)	96 (84.2)		173 (42.3)	226 (75.3)	
GLDC in tumor			0.540			0.114			0.072
Negative	365 (52.7)	5 (31.3)		322 (54.1)	48 (42.1)		230 (56.2)	140 (46.7)	
Positive	328 (47.3)	11 (68.8)		273 (45.9)	66 (57.9)		179 (43.8)	160 (53.3)	
GLDC in stroma			**<0.001**			**<0.001**			**<0.001**
Negative	614 (88.6)	7 (43.8)		552 (92.8)	69 (60.5)		387 (94.6)	234 (78.0)	
Positive	79 (11.4)	9 (56.3)		43 (7.2)	45 (39.5)		22 (5.4)	66 (22.0)	

*p-value was corrected by the Bonferroni method.

### Impact of serine-/glycine-metabolism–associated enzymes on patients' prognosis

**Figure 5:**
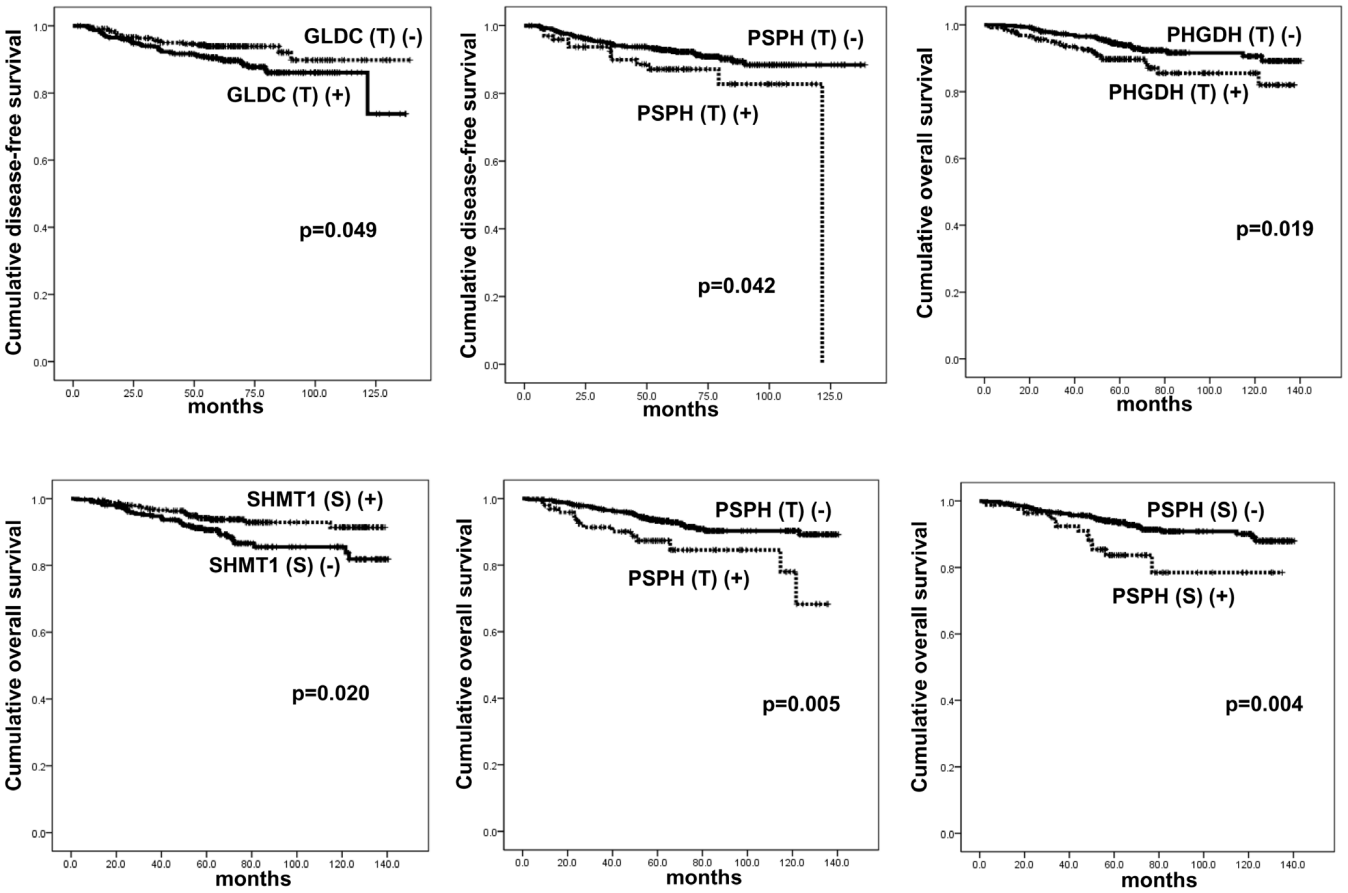
Disease-free and overall survival according to the expression of enzymes associated with serine/glycine metabolism.

Statistics was used to assess the effect of the expression of the metabolism-associated enzymes on patients' survival and prognostic parameters. By univariate analysis, tumoral PSPH positivity (p = 0.042) and tumoral GLDC negativity (p = 0.049) were associated with short disease-free survival (DFS) and tumoral PHGDH positivity (p = 0.019), tumoral PSPH positivity (p = 0.005), stromal PSPH positivity (p = 0.004), and stromal SHMT1 negativity (p = 0.020) were associated with short overall survival (OS) (Table 8 and Figure 5).

**Table 8:** Impact of expression of enzymes associated with serine/glycine metabolism on disease-free and overall survival tested by log-rank analysis.

Parameters	Number of patients/recurrence/death	Disease-free survival		Overall survival	
		Mean survival (95% CI) months	*P*-value	Mean survival (95% CI) months	*P*-value
PHGDH in tumor			0.137		**0.019**
Negative	452/36/31	128 (125–132)		132 (129–135)	
Positive	257/27/28	120 (113–127)		123 (118–128)	
PSAT1 in tumor			0.507		0.225
Negative	526/43/38	126 (123–129)		129 (126–132)	
Positive	183/20/21	124 (118–130)		127 (122–132)	
PSPH in tumor			**0.042**		**0.005**
Negative	611/50/45	128 (125–131)		131 (128–133)	
Positive	98/13/14	107 (99–115)		117 (108–126)	
PSPH in stroma			0.097		**0.004**
Negative	625/52/46	126 (122–130)		131 (128–133)	
Positive	84/11/13	117 (107–127)		116 (106–125)	
SHMT1 in tumor			0.208		0.091
Negative	598/50/46	127 (124–130)		130 (127–133)	
Positive	111/13/13	121 (112–130)		125 (117–132)	
SHMT1 in stroma			0.107		**0.020**
Negative	310/34/35	121 (115–127)		126 (121–130)	
Positive	399/29/24	128 (124–132)		131 (128–134)	
GLDC in tumor			**0.049**		0.377
Negative	370/42/36	122 (116–127)		129 (125–132)	
Positive	339/21/23	130 (126–134)		131 (127–135)	
GLDC in stroma			0.568		0.951
Negative	621/54/52	126 (122–130)		130 (127–132)	
Positive	88/9/7	115 (106–124)		123 (116–130)	

By multivariate Cox analysis, high T staging (T1 vs. T2/3, hazard ratio: 2.245, 95% CI: 1.212–4.160, p = 0.01) and lymph-node metastasis (hazard ratio: 2.574, 95% CI: 1.513–4.379, p<0.001) were associated with short DFS (Table 9). Meanwhile, lymph-node metastasis (hazard ratio: 2.204, 95% CI: 1.284–3.782, p = 0.004), tumoral PSPH positivity (hazard ratio: 2.068, 95% CI: 1.049–4.079, p = 0.036), stromal PSPH positivity (hazard ratio: 2.152, 95% CI: 1.107–4.184, p = 0.024), and stromal SHMT1 negativity (hazard ratio: 2.142, 95% CI: 1.219–3.764, p = 0.008) were associated with short OS.

**Table 9:** Multivariate analysis of breast cancer survival.

Included parameters	Disease-free survival			Overall survival		
	Hazard ratio	95% CI	*P*-value	Hazard ratio	95% CI	*P*-value
T stage			**0.010**			0.101
T1 versus T2–3	2.245	1.212–4.160		1.656	0.906–3.024	
N stage			**<0.001**			**0.004**
N0 versus N1–3	2.574	1.513–4.379		2.204	1.284–3.782	
Histologic grade			0.271			0.990
I/II versus III	1.365	0.785–2.374		1.004	0.562–1.791	
ER status			0.129			0.322
Negative versus Positive	1.849	0.836–4.089		1.495	0.675–3.311	
PR status			1.298			0.141
Negative versus Positive	1.298	0.595–2.833		1.839	0.817–4.140	
HER-2 status			0.097			0.112
Negative versus Positive	1.645	0.913–2.962		1.638	0.891–3.012	
PHGDH in tumor			0.367			0.932
Negative versus Positive	0.754	0.409–1.393		0.973	0.520–1.819	
PSPH in tumor			0.069			**0.036**
Negative versus Positive	1.911	0.952–3.836		2.068	1.049–4.079	
PSPH in stroma			0.399			**0.024**
Negative versus Positive	1.345	0.676–2.677		2.152	1.107–4.184	
SHMT1 in stroma			0.141			**0.008**
Negative versus Positive	1.490	0.876–2.532		2.142	1.219–3.764	
GLDC in tumor			1.654			0.634
Negative versus Positive	1.654	0.935–2.925		1.149	0.649–2.033	

Next, we examined the correlation between the expression of the enzymes associated with serine/glycine metabolism and survival of breast cancer patients by molecular subtypes. By univariate analysis, patients with each molecular subtype showed unfavorable OS in accordance with expression profiles of the following proteins (Table S1 in File S1): tumoral PHGDH positivity (p = 0.033), tumoral PSPH positivity (p = 0.020), and stromal SHMT1 negativity (p = 0.026) in the luminal-A; stromal PSPH positivity (p = 0.041) in the HER-2; and stromal SHMT1 negativity (p = 0.040) in the TNBC subtype. In addition, tumoral PSPH positivity (p = 0.042) and stromal SHMT1 negativity (p = 0.009) in the TNBC subtype were associated with unfavorable DFS.

By multivariate Cox analysis (Tabel S2), tumoral PSPH positivity (hazard ratio: 7.067, 95% CI: 1.316–37.95, p = 0.023) and stromal SHMT1 negativity (hazard ratio: 5.300, 95% CI: 1.101–25.50, p = 0.037) were associated with short OS in the luminal-A subtype; lymph-node metastasis (hazard ratio: 17.51, 95% CI: 1.969–155.8, p = 0.010) was correlated with short DFS, and lymph-node metastasis (hazard ratio: 21.49, 95% CI: 2.434–189.7, p = 0.006) and ER negativity (hazard ratio: 14.38, 95% CI: 1.171–176.7, p = 0.037) were correlated with short OS in the luminal-B subtype; lymph-node metastasis (hazard ratio: 6.456, 95% CI: 2.376–17.54, p<0.001), tumoral PHGDH negativity (hazard ratio: 3.358, 95% CI: 1.448–8.866, p = 0.006), tumoral PSPH positivity (hazard ratio: 6.173, 95% CI: 2.323–16.40, p<0.001), stromal SHMT1 negativity (hazard ratio: 3.312, 95% CI: 1.256–8.736, p = 0.016), and tumoral GLDC negativity (hazard ratio: 4.231, 95% CI: 1.384–12.92, p = 0.011) correlated with short DFS in TNBC subtype. In addition, lymph-node metastasis (hazard ratio: 2.799, 95% CI: 1.119–7.002, p = 0.028), tumoral PHGDH negativity (hazard ratio: 3.624, 95% CI: 1.500–8.757, p = 0.004), tumoral PSPH positivity (hazard ratio: 3.880, 95% CI: 1.495–10.07, p = 0.005), and stromal SHMT1 negativity (hazard ratio: 2.605, 95% CI: 1.024–6.626, p = 0.044) correlated with short OS in TNBC subtype.

## Discussion

In this study, we examined differential expression patterns of some of the enzymes associated with serine/glycine metabolism in the different molecular subtypes of the breast cancer in separate histopathological tumor compartments (i.e., cancer cell and tumor stroma) and investigated their likely clinical implications. Enzymes associated with serine metabolism, including PHGDH and PSPH were highly expressed in the TNBC cell lines and tissues, but not significantly expressed in the luminal-A subtype. Previous studies have reported increased expression of PHGDH especially in the ER-negative cancers, showing an expression rate about 70% [3]. We also confirmed that PHGDH was highly expressed in the TNBC subtype, representative ER-negative breast cancers. PHGDH was expressed in 68.1% of the ER-negative cancers in comparison with 18.5% in the ER-positive cancers and was associated with high histologic grading, ER negativity, PR negativity, and high Ki-67 LI, which are known as poor prognosticators. Similar to PHGDH, PSPH was frequently expressed in the TNBC subtype and correlated with high histopathological grading, ER negativity, PR negativity, and high Ki-67 LI. Thus, we hypothesize that a breast cancer subset with high expression of enzymes associated with serine metabolism (PHGDH and PSPH) could present with an aggressive behavior. It is likely that metabolic demand for serine metabolites may increase as the overall metabolic demand increases in breast cancers with aggressive pathological behavior.

Previous reports demonstrated that the glycolysis-associated enzymes, such as Glut-1, CA9, and MCT4, were distinctly expressed in different molecular subtypes: high in the TNBC and basal-like, but low in the luminal-A subtype [9], [10]. This study showed that expression of Glut-1 and CA9 were linked to PHGDH and PSPH expression, which could be explained because serine metabolites serve as glycolytic intermediates. Thus, serine metabolism provides α-ketoglutarate, a TCA-cycle intermediate, to cancer cells instead of serine itself, leading to acceleration of mitochondrial metabolism using excess α-ketoglutarate by highly expressed enzymes which drive mitochondrial energy metabolism [3].

Similar to PHGDH and PSPH, SHMT1 was highly expressed in the TNBC subtype and lowest in the luminal-A subtype. As previously reported, glycine metabolism is key in accelerating cancerous cell proliferation; we believe that high SHMT1 levels in the TNBC subtype ensures high proliferative capacity, showing the highest Ki-67 LI among other molecular subtypes [5].

GLDC was also expressed at differing levels in different molecular subtypes of breast cancer. The highest GLDC expression was found in the HER-2 and the lowest in the TNBC subtype, in contrast to enzymes involved in the serine metabolism. High GLDC levels have been reported in several human cancers, including non-small-cell lung carcinoma, ovarian cancer, and germ-cell tumors; however, GLDC levels have not been studied in breast cancer [4]. The precise mechanism of increased GLDC in HER-2 subtype could not be postulated, but GLDC levels were previously examined in MCF10A cells after oncogenic transformation by KRAS^G12D^, PIK3CA^E545K^, or MYC^T58A^. All three oncogenes increased GLDC expression by 20-fold, suggesting that oncogene-induced GLDC transcription can commonly be driven by oncogenic Ras, PI3K, and Myc [4]. Therefore, we hypothesize that the TNBC subtype, which is not associated with any well-known driving oncogene, revealed the lowest GLDC level, unlike the HER-2 subtype driven by certain HER-2 oncogenes.

Appreciably, breast cancer stromal components expressed elevated PSPH, SHMT1, and GLDC levels. According to the “reverse Warburg effect” theory, both the stromal and epithelial tumor components are involved in cancer metabolism: oxidative phosphorylation by the functional mitochondria occurs in the epithelial tumor component; glycolysis due to increased autophagic activity leads to dysfunctional mitochondria in the stromal component [18], [19], [20], [21]. Similar to the epithelial tumor component, the stromal component expressed the highest levels of PSPH, SHMT1, and GLDC in the HER-2 and lowest in TNBC subtype. Expression of these enzymes was statistically correlated with expression of glycolysis-associated enzymes such as Glut-1, CA9, and MCT-4, supporting the possibility of serine/glycine metabolism occurring in the tumor stroma. Although the exact mechanisms underlying high levels of the enzymes associated with serine/glycine metabolism in the HER-2 subtype are unknown, the glutaminolysis-associated enzymes, including glutaminase 1 (GLS1), glutamate dehydrogenase (GDH), and amino acid transporter 2 (ASCT2), have been highly expressed in the HER-2 subtype, suggesting that tumor stromal cells in this subtype are active metabolically [11].

We also examined the potential clinical implications of the expression profiles metabolism-associated enzymes in the different molecular subtypes of breast cancer. Particularly, expression of enzymes associated with serine/glycine metabolism was correlated with patients' prognosis. In the entire patient population irrespective of subtype, tumoral PSPH positivity, stromal PSPH positivity, and stromal SHMT1 negativity were independent factors for poor prognosis. According to the molecular subtype, tumoral PSPH positivity and stromal SHMT1 negativity in the luminal-A subtype and tumoral PHGDH negativity, tumoral PSPH positivity, and stromal SHMT1 negativity in the TNBC subtype were independent factors for poor prognosis. Although correlations between levels of enzymes associated with serine/glycine metabolism and tumor prognosis have remained unknown, several previous studies have shown that high levels of glycolysis-associated enzymes, including Glut-1 and CA9, were correlated with unfavorable prognosis in breast cancer [9], [22], [23].

Although high SHMT2, but not SHMT1, was correlated with breast cancer patient mortality [5], we found that stromal SHMT1 negativity was associated with poor prognosis. Similarly, tumoral PHGDH negativity in the TNBC subtype was correlated with poor prognosis. However, further studies are needed to confirm these findings. In conclusion, we identified differential expression of the enzymes associated with serine/glycine metabolism according to breast cancer molecular subtypes. The highest expression of enzymes associated with serine metabolism was found in the TNBC subtype, and the lowest, in the luminal-A subtype. Expression of enzymes associated with glycine metabolism was high in the HER-2 type in both tumor and stromal compartments.

## Supporting Information

File S1 **Tables S1 and S2.**
(DOC)
