# Supplementary material for: Differential Expression of Enzymes Associated with Serine/Glycine Metabolism in Different Breast Cancer Subtypes
Source: PLoS One. 2014 Jun 30;9(6):e101004. doi: 10.1371/journal.pone.0101004 (PMC4076239; doi:10.1371/journal.pone.0101004)
Supplement: File S1 — Tables S1 and S2. (DOC) [file pone.0101004.s001.doc]

| **Table S1.** Univariate analysis of the impact for the expression of serine/glycine metabolism-related proteins in breast cancers on disease-free survival or overall survival according to the molecular subtype | | | | | | | | | | | | | | | | |
| --- | --- | --- | --- | --- | --- | --- | --- | --- | --- | --- | --- | --- | --- | --- | --- | --- |
| Parameters | Luminal A | | | | Luminal B | | | | HER-2 | | | | TNBC | | | |
|  | Disease-free survival | | Overall survival | | Disease-free survival | | Overall survival | | Disease-free survival | | Overall survival | | Disease-free survival | | Overall survival | |
|  | Mean survival  (95% CI) months | *P* -value | Mean survival  (95% CI) months | *P* -value | Mean survival  (95% CI) months | *P* -value | Mean survival  (95% CI) months | *P* -value | Mean survival  (95% CI) months | *P* -value | Mean survival  (95% CI) months | *P* -value | Mean survival  (95% CI) months | *P* -value | Mean survival  (95% CI) months | *P* -value |
| PHGDH in tumor |  | 0.873 |  | **0.033** |  | 0.387 |  | 0.852 |  | 0.234 |  | 0.164 |  | 0.111 |  | 0.051 |
| Negative | 130 (126-134) |  | 136 (133-139) |  | 128 (122-134) |  | 130 (124-135) |  | 117 (105-129) |  | 127 (114-139) |  | 96 (84-108) |  | 111 (97-126) |  |
| Positive | 103 (97-109) |  | 126 (116-136) |  | 112 (102-121) |  | 123 (110-137) |  | 86  (74-98) |  | 107 (92-122) |  | 120 (111-128) |  | 124 (117-131) |  |
| PSAT1 in tumor |  | 0.630 |  | 0.924 |  | 0.339 |  | 0.134 |  | 0.241 |  | 0.253 |  | 0.274 |  | 0.308 |
| Negative | 120 (116-124) |  | 134 (130-137) |  | 114 (110-119) |  | 130 (125-135) |  | 79 (69-88) |  | 104 (92-117) |  | 122 (115-129) |  | 122 (115-130) |  |
| Positive | 132 (126-137) |  | 135 (130-141) |  | 124 (111-136) |  | 120 (107-134) |  | 118 (105-131) |  | 128 (115-140) |  | 106 (94-119) |  | 118 (106-130) |  |
| PSPH in tumor |  | n/a |  | **0.020** |  | 0.744 |  | 0.759 |  | n/a |  | n/a |  | **0.042** |  | 0.054 |
| Negative | n/a |  | 135 (133-138) |  | 129 (123-134) |  | 129 (124-135) |  | n/a |  | n/a |  | 124 (117-131) |  | 125 (118-132) |  |
| Positive | n/a |  | 105 (84-125) |  | 109 (93-126) |  | 128 (117-139) |  | n/a |  | n/a |  | 100 (88-112) |  | 110 (97-123) |  |
| PSPH in stroma |  | n/a |  | n/a |  | 0.427 |  | 0.054 |  | 0.313 |  | **0.041** |  | 0.875 |  | 0.988 |
| Negative | n/a |  | n/a |  | 130 (125-135) |  | 131 (126-136) |  | 95 (86-104) |  | 125 (115-135) |  | 116 (107-125) |  | 121 (114-128) |  |
| Positive | n/a |  | n/a |  | 106 (92-119) |  | 111 (95-128) |  | 100 (75-125) |  | 95 (70-120) |  | 120 (106-133) |  | 121 (108-133) |  |
| SHMT1 in tumor |  | n/a |  | n/a |  | 0.982 |  | 0.745 |  | 0.393 |  | 0.356 |  | 0.688 |  | 0.399 |
| Negative | n/a |  | n/a |  | 128 (123-134) |  | 129 (123-135) |  | 89 (80-99) |  | 111 (100-122) |  | 118 (110-126) |  | 123 (115-131) |  |
| Positive | n/a |  | n/a |  | 89 (80-99) |  | 113 (101-125) |  | 121 (105-138) |  | 131 (114-148) |  | 116 (105-128) |  | 117 (106-128) |  |
| SHMT1 in stroma |  | 0.925 |  | **0.026** |  | 0.512 |  | 0.490 |  | 0.785 |  | 0.834 |  | **0.009** |  | **0.040** |
| Negative | 131 (127-135) |  | 131 (126-137) |  | 110 (102-117) |  | 126 (115-137) |  | 91 (76-106) |  | 119 (100-137) |  | 106 (95-118) |  | 113 (103-123) |  |
| Positive | 122 (116-127) |  | 136 (134-138) |  | 129 (123-136) |  | 130 (124-136) |  | 112 (100-123) |  | 114 (102-125) |  | 129 (123-135) |  | 129 (122-135) |  |
| GLDC in tumor |  | 0.331 |  | 0.998 |  | 0.213 |  | 0.375 |  | 0.644 |  | 0.328 |  | 0.321 |  | 0.877 |
| Negative | 130 (125-134) |  | 135 (131-139) |  | 108 (102-114) |  | 128 (121-135) |  | 90 (75-105) |  | 111 (93-128) |  | 114 (104-124) |  | 121 (114-129) |  |
| Positive | 123 (117-128) |  | 133 (130-137) |  | 134 (129-139) |  | 130 (120-140) |  | 112 (100-124) |  | 124 (112-136) |  | 118 (109-126) |  | 118 (108-128) |  |
| GLDC in stroma |  | 0.125 |  | 0.974 |  | 0.637 |  | 0.346 |  | 0.461 |  | 0.442 |  | 0.714 |  | 0.760 |
| Negative | 131 (128-135) |  | 135 (132-138) |  | 130 (124-135) |  | 131 (125-136) |  | 108 (96-120) |  | 117 (105-130) |  | 117 (109-125) |  | 121 (114-127) |  |
| Positive | 115 (100-129) |  | 129 (121-137) |  | 96 (86-105) |  | 116 (101-130) |  | 97 (84-109) |  | 120 (105-135) |  | 65 (57-73) |  | 113 (99-128) |  |

| **Table S2.** Multivariate analysis for breast-cancer survival according to the molecular subtype | | | | | | | | | | | | | | | | | | | | | | | | |
| --- | --- | --- | --- | --- | --- | --- | --- | --- | --- | --- | --- | --- | --- | --- | --- | --- | --- | --- | --- | --- | --- | --- | --- | --- |
| Included parameters | Luminal A | | | | | | Luminal B | | | | | | HER-2 | | | | | | TNBC | | | | | |
|  | Disease-free survival | | | Overall survival | | | Disease-free survival | | | Overall survival | | | Disease-free survival | | | Overall survival | | | Disease-free survival | | | Overall survival | | |
|  | HR | 95%CI | *P* | HR | 95%CI | *P* | HR | 95%CI | *P* | HR | 95%CI | *P* | HR | 95%CI | *P* | HR | 95%CI | *P* | HR | 95%CI | *P* | HR | 95%CI | *P* |
| T stage |  |  | 0.772 |  |  | 0.873 |  |  | 0.130 |  |  | 0.813 |  |  | 0.132 |  |  | 0.450 |  |  | 0.435 |  |  | 0.163 |
| T1 vs. T2-3 | 1.175 | 0.393-3.511 |  | 0.907 | 0.276-2.983 |  | 3.304 | 0.703-15.53 |  | 1.185 | 0.290-4.852 |  | 3.775 | 0.669-21.29 |  | 1.944 | 0.346-10.92 |  | 1.605 | 0.489-5.268 |  | 2.268 | 0.719-7.156 |  |
| N stage |  |  | 0.524 |  |  | 0.935 |  |  | **0.010** |  |  | **0.006** |  |  | 0.446 |  |  | 0.592 |  |  | **<0.001** |  |  | **0.028** |
| N0 vs. N1-3 | 1.417 | 0.486-4.132 |  | 1.050 | 0.325-3.390 |  | 17.51 | 1.969-155.8 |  | 21.49 | 2.434-189.7 |  | 0.606 | 0.166-2.203 |  | 1.406 | 0.404-4.889 |  | 6.456 | 2.376-17.54 |  | 2.799 | 1.119-7.002 |  |
| Histologic grade |  |  | 0.143 |  |  | 0.367 |  |  | 1.277 |  |  | 0.938 |  |  | 0.742 |  |  | 0.457 |  |  | 0.651 |  |  | 0.353 |
| I/II vs. III | 2.672 | 0.718-9.951 |  | 2.100 | 0.419-10.51 |  | 1.277 | 0.399-4.086 |  | 1.052 | 0.293-3.782 |  | 1.220 | 0.373-3.995 |  | 1.633 | 0.448-5.951 |  | 1.231 | 0.500-3.034 |  | 0.666 | 0.282-1.572 |  |
| ER status |  |  | n/a |  |  | n/a |  |  | 0.074 |  |  | **0.037** |  |  | n/a |  |  | n/a |  |  | n/a |  |  | n/a |
| (-) vs. (+) | n/a | n/a |  | n/a | n/a |  | 8.186 | 0.816-82.21 |  | 14.38 | 1.171-176.7 |  | n/a | n/a |  | n/a | n/a |  | n/a | n/a |  | n/a | n/a |  |
| PR status |  |  | 0.991 |  |  | 0.502 |  |  | 0.608 |  |  | 0.209 |  |  | n/a |  |  | n/a |  |  | n/a |  |  | n/a |
| (-) vs. (+) | 1.008 | 0.267-3.799 |  | 1.605 | 0.403-6.391 |  | 1.387 | 0.397-4.847 |  | 2.336 | 0.621-8.786 |  | n/a | n/a |  | n/a | n/a |  | n/a | n/a |  | n/a | n/a |  |
| HER-2 status |  |  | n/a |  |  | n/a |  |  | 0.360 |  |  | 0.569 |  |  | n/a |  |  | n/a |  |  | n/a |  |  | n/a |
| (-) vs. (+) | n/a | n/a |  | n/a | n/a |  | 1.767 | 0.523-5.968 |  | 1.461 | 0.369-5.394 |  | n/a | n/a |  | n/a | n/a |  | n/a | n/a |  | n/a | n/a |  |
| PHGDH (T) |  |  | 0.790 |  |  | 0.082 |  |  | 0.274 |  |  | 0.565 |  |  | 0.068 |  |  | 0.057 |  |  | **0.006** |  |  | **0.004** |
| (-) vs. (+) | 1.233 | 0.265-5.740 |  | 3.107 | 0.865-11.16 |  | 0.334 | 0.047-2.384 |  | 0.879 | 0.167-21.68 |  | 3.234 | 0.915-11.42 |  | 3.414 | 0.965-12.07 |  | 3.358 | 1.448-8.866 |  | 3.624 | 1.500-8.757 |  |
| PSPH (T) |  |  | n/a |  |  | **0.023** |  |  | 0.355 |  |  | 0.565 |  |  | n/a |  |  | n/a |  |  | **<0.001** |  |  | **0.005** |
| (-) vs. (+) | n/a | n/a |  | 7.067 | 1.316-37.95 |  | 3.424 | 0.253-46.37 |  | 2.010 | 0.186-21.68 |  | n/a | n/a |  | n/a | n/a |  | 6.173 | 2.323-16.40 |  | 3.880 | 1.495-10.07 |  |
| PSPH (S) |  |  | n/a |  |  | n/a |  |  | 0.119 |  |  | 0.006 |  |  | 0.981 |  |  | 0.129 |  |  | 0.671 |  |  | 0.901 |
| (-) vs. (+) | n/a | n/a |  | n/a | n/a |  | 3.439 | 0.726-16.28 |  | 11.88 | 2.056-68.66 |  | 1.020 | 0.205-5.084 |  | 3.782 | 0.678-21.09 |  | 0.781 | 0.250-2.443 |  | 1.076 | 0.340-3.406 |  |
| SHMT1 (S) |  |  | 0.854 |  |  | **0.037** |  |  | 0.417 |  |  | 0.164 |  |  | 0.782 |  |  | 0.276 |  |  | **0.016** |  |  | **0.044** |
| (-) vs. (+) | 0.902 | 0.300-2.711 |  | 5.300 | 1.101-25.50 |  | 1.763 | 0.449-6.925 |  | 3.001 | 0.637-14.12 |  | 1.241 | 0.269-5.727 |  | 2.555 | 0.472-13.83 |  | 3.312 | 1.256-8.736 |  | 2.605 | 1.024-6.626 |  |
| GLDC (T) |  |  | 0.366 |  |  | 0.969 |  |  | 0.194 |  |  | 0.594 |  |  | 0.785 |  |  | 0.954 |  |  | **0.011** |  |  | 0.367 |
| (-) vs. (+) | 1.715 | 0.533-5.520 |  | 1.026 | 0.291-3.617 |  | 2.728 | 0.600-12.41 |  | 1.497 | 0.339-6.610 |  | 0.844 | 0.251-2.844 |  | 0.966 | 0.293-3.183 |  | 4.231 | 1.384-12.92 |  | 1.603 | 0.575-4.470 |  |

(T), tumor, (s), stroma
